# Correlates of Cancer-Related Fatigue among Colorectal Cancer Patients Undergoing Postoperative Adjuvant Therapy Based on the Theory of Unpleasant Symptoms

**DOI:** 10.3390/curroncol29120720

**Published:** 2022-11-26

**Authors:** Song Wang, Ning Jiang, Yuanyuan Song, Lihua Ma, Ying Niu, Jing Song, Xiaolian Jiang

**Affiliations:** 1 West China School of Nursing/West China Hospital, Sichuan University, Chengdu 610041, China; 2 Department of Critical Care Medicine, West China Hospital of Sichuan University, Chengdu 610041, China; 3 Department of Endocrinology, The First Hospital of Lanzhou University, Lanzhou 730013, China; 4 School of Nursing, Xinxiang Medical University, Xinxiang 453003, China; 5 School of Stomatology, Bengbu Medical College, Bengbu 233030, China

**Keywords:** colorectal cancer, adjuvant therapy, symptom management, cancer-related fatigue, psycho-oncology, social support, hope, self-disclosure

## Abstract

Background: Cancer-related fatigue (CRF) is a common and burdensome symptom in cancer patients that is influenced by multiple factors. Identifying factors associated with CRF may help in developing tailored interventions for fatigue management. This study aimed to examine the correlates of CRF among colorectal cancer patients undergoing postoperative adjuvant therapy based on the theory of unpleasant symptoms. Methods: A cross-sectional study was implemented, and finally, a sample of 363 participants from one tertiary general hospital and one tertiary cancer hospital was purposively recruited. Data were collected using the general information questionnaire, cancer fatigue scale, the distress disclosure index, Herth hope index, and perceived social support scale. Univariate analysis and multiple linear regression analysis were performed to determine the correlates of CRF. Results: The mean score of CRF among colorectal cancer patients was 21.61 (SD = 6.16, 95% CI 20.98–22.25), and the fatigue degree rating was “moderate”. The multiple linear regression model revealed that 49.1% of the variance in CRF was explained by hope, sleep disorder, internal family support, self-disclosure, pain, and time since operation. Conclusions: Our study identified several significant, modifiable factors (self-disclosure, hope, internal family support, pain, and sleep disorder) associated with CRF. Understanding these correlates and developing targeted psychosocial interventions may be associated with the improvement of CRF in patients with colorectal cancer.

## 1. Introduction

Colorectal cancer is the most common malignant tumor of the digestive tract [1]. According to the GLOBOCAN 2020 statistics from the International Agency for Research on Cancer (IARC), there were an estimated 1.93 million new cases of colorectal cancer, and 935,000 deaths from colorectal cancer occurred around the world [2]. The incidence and mortality of colorectal cancer ranked the third and the second of all cancers worldwide, respectively. Meanwhile, there were an estimated 560,000 new colorectal cancer cases, and 286,000 colorectal cancer deaths occurred in China, the incidence and mortality of colorectal cancer accounted for the second and the fifth of all malignancies, respectively [3]. With increasing incidence and mortality, colorectal cancer has imposed a huge disease burden to the current society and turned into a growing global public health challenge [4].

With the continuous progress and development of cancer diagnosis and treatment technology, the overall survival of colorectal cancer has been prolonged. There are many treatment options for colorectal cancer, including surgery, chemotherapy, radiotherapy, targeted therapy, immunotherapy, traditional Chinese medicine (TCM) treatment, palliative care, and so on [5,6]. These associated treatments not only lead to good prospects for patients with colorectal cancer but also bring multiple symptom burdens, which negatively affect their quality of life [7]. Therefore, healthcare professionals should also focus on symptom management and quality of life of patients with colorectal cancer in addition to prevention and treatment of the disease.

Patients diagnosed with cancer frequently experience a series of complicated symptoms throughout all phases of the illness and treatment (e.g., pain, sleep disturbance, tiredness, psychological stress, cognitive impairment, nausea and vomiting). Cancer-related fatigue (CRF) is the most common and distressing symptom, especially in cancer patients receiving chemotherapy, radiotherapy, and biological therapy [8]. According to the definition proposed by the National Comprehensive Cancer Network (NCCN), CRF was defined as “a distressing, persistent, subjective sense of physical, emotional, and/or cognitive tiredness or exhaustion related to cancer or cancer treatment that is not proportional to recent activity and interferes with usual functioning” [9]. The prevalence of CRF varies in different cancer populations. The pooled prevalence of CRF among mixed cancer patients was estimated to be 52% in a meta-analysis of 84 studies [10]. Vardy [11] found that the incidence of CRF ranged from 57% to 70% in patients with colorectal cancer. An observational study revealed that the prevalence of CRF was up to 100% among colorectal cancer patients after fast-track surgery [12]. CRF is different from the general fatigue caused by physical activity. It is more severe and troubling, persists for a long time, and cannot be effectively relieved by rest or sleep [13]. CRF has detrimental effects on physical, psychosocial functioning, and economic consequences for both patients and their primary caregivers [14], and it may undermine quality of life, impact treatment compliance, and reduce the overall survival of patients [15,16,17].

Despite the high prevalence and negative effects of CRF, such a symptom has been poorly managed in clinical practice [18,19,20]. One of the main obstacles to the effective management may be related to a lack of information about the correlates of CRF. Therefore, understanding and identifying factors associated with CRF is important in developing a targeted management strategy to attenuate this symptom for cancer patients. The correlates of CRF are complex and multifactorial, involving sociodemographic, tumor-related, medical, psychosocial, behavioral, and physiological factors [21,22]. Although a wealth of studies on CRF have been conducted, previous studies on the influencing factors of CRF mainly focused on the survivors with lung cancer and breast cancer; there was limited evidences regarding the correlates of CRF among colorectal cancer patients. Moreover, the associations between CRF and its predictive factors, such as sex, age, anxiety, depression, social support, and other variables, have not been consistently identified and remain controversial [10,16,23,24,25,26,27,28]. These existing problems limit the development of tailored interventions for this population to some extent.

**Figure 1:**
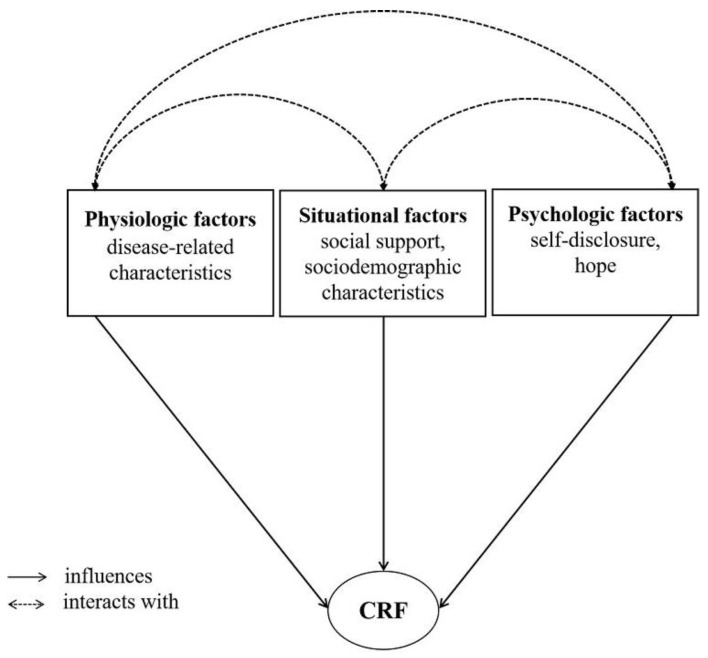
The hypothesized model on influencing factors of CRF in patients with colorectal cancer. Note: CRF, cancer-related fatigue.

As far as we know, due to a lack of theoretical framework as guidance, the majority of current research mainly explores the related factors of CRF from a single perspective (e.g., physiologic or psychologic). Given the multidimensional nature of CRF, using a comprehensive assessment of CRF is recommended. The theory of unpleasant symptoms (TUS) was developed and updated by Lenz et al. [29] in 1995 and 1997, consisting of three components: symptoms, their contributing factors and performance. The theory holds that the factors leading to unpleasant symptoms include physiologic, psychologic, and situational factors. The physiologic factors include normal systems, pathologic problems (e.g., disease-related characteristics), and energy level; the psychologic factors include mental state, affective reactions to the illness (e.g., hope, self-disclosure), etc.; while the situational factors include the aspects of social and physical environment, such as social support, sociodemographic characteristics, etc. [29]. TUS has been used to examine the influencing factors of CRF in lung cancer patients and develop some tailored interventions for the treatment and management of this symptom [30]. However, studies into the correlates of CRF among colorectal cancer patients based on the TUS are rare, and some psychosocial factors can be modified. Social support, hope, and self-disclosure are major positive psychosocial concepts, which are widely used in supportive care of cancer patients. Many researchers found evidence that social support, hope, and self-disclosure played positive roles in the physical and mental health of cancer patients [31,32]. However, the effects of these psychosocial factors on CRF in colorectal cancer patients have rarely been examined before, or the conclusions are still discrepant. In addition, two literature reviews reported that the relationships between CRF and some sociodemographic characteristics (e.g., level of income, sex, age) or disease-related characteristics (e.g., therapeutic method, insomnia, pain) were still unclear [8,10]; the findings were mixed. Therefore, the present study constructed a preliminary hypothesized theoretical model based on the TUS (see Figure 1), and it analyzed the correlates of CRF among colorectal cancer patients from three aspects of physiologic, psychologic, and situational factors. We aimed to determine the physiological psychosocial correlates of CRF in colorectal cancer patients undergoing postoperative adjuvant therapy, and in particular, test the effects of controllable positive psychosocial factors (social support, self-disclosure, hope) on CRF, help design effective interventions and facilitate CRF management for this population.

## 2. Methods

### 2.1. Study Design and Sample

This was a cross-sectional survey with a convenience sample of 363 colorectal cancer patients undergoing postoperative adjuvant therapy, who were recruited from July 2020 to February 2021 in one tertiary general hospital and one tertiary cancer hospital located in Bengbu, Anhui, China. G*Power (version 3.1.9.2) software was used for the sample size calculation, when the medium effect size of multiple regression analysis for the F-test was 0.15, the α value was 0.05, the power (1-β) was 0.90 [33], the number of predictors was 18, and the minimum sample size of this study should be 183, so the number of patients included in our study met the requirements.

The cases were included if they met the following inclusion criteria: (a) patients diagnosed with colorectal cancer by pathological examination or histological examination; (b) age ≥ 18 years; (c) Karnofsky performance status (KPS) score ≥ 60 points; (d) being aware of colorectal cancer diagnosis and receiving postoperative adjuvant therapy; and (e) signing the informed consent and willing to participate in this study.

The cases with the following conditions were excluded: (a) patients within 2–4 weeks since operation; (b) with primary tumors in other sites; (c) with serious heart, liver, lung, kidney, and other diseases or comorbidities; (d) having received neoadjuvant therapy before operation; (e) language/written communication difficulties; (f) with cognitive dysfunction, or a history of mental illness; and (g) unwilling to participate in the study.

This study was reported in compliance with the STROBE checklist for cross-sectional studies (see Supplementary Table S1).

### 2.2. Procedures

The study was approved by the Biomedical Research Ethics Review Committee of the relevant hospitals (No: 2020-482), and informed consent was obtained from all participants before data collection. Researchers screened eligible patients through the hospital information system (HIS) and/or medical records and sincerely invited them to this study. The purpose and method of the study were introduced to them. After signing the informed consent, the patients were asked to fill out the self-reported questionnaires either offline or online through ‘wenjuanxing’ (a widely used online investigation platform in China Mainland) [34]. Each participant took 15–20 min to complete the questionnaires. The investigators checked the questionnaires for completeness and instructed patients to fill in any missing information.

### 2.3. Measures

#### 2.3.1. General Information Questionnaire

General information includes sociodemographic and colorectal cancer-related characteristics, such as sex, age, body mass index (BMI), educational level, marital status, family per capita monthly income, and occupational status; cancer type, clinical stage, time since operation, treatment methods, number of chemotherapy cycles, pain, and sleep disorder.

#### 2.3.2. Cancer Fatigue Scale (CFS)

CFS was developed by Okuyama [35] to evaluate the fatigue symptom of cancer patients, including 15 items and 3 dimensions: physical fatigue (items 1, 2, 3, 6, 9, 12, and 15), emotional fatigue (items 5, 8, 11, and 14, scored reversely), and cognitive fatigue (items 4, 7, 10, and 13). A Likert five-point rating method was used for each item, with 0 (not at all)~4 (very much), the total score of physical fatigue is 0~28 points, the total score of emotional fatigue and cognitive fatigue is 0~16 points, respectively, and the total score of CFS is 0~60 points. Here, 0 means no fatigue; the higher the score, the more severe the fatigue (1~20 means mild fatigue; 21~40 means moderate fatigue; and 41~60 means severe fatigue). The scale had good reliability and validity in 307 patients with mixed types of cancer, Cronbach’s α coefficients of physical fatigue, emotional fatigue, cognitive fatigue, and total fatigue were 0.89, 0.79, 0.79, and 0.88, respectively. The Chinese version of CFS has also been proven to have good reliability and validity within the Chinese populations [36,37]. In the present sample, the Cronbach’s α coefficient for this scale among colorectal cancer patients was 0.93.

#### 2.3.3. The Distress Disclosure Index (DDI)

DDI is a 12-item inventory designed by Kahn [38] to assess the degree to which an individual discloses his/her personal privacy such as troubles and distress experiences to others. A five-point Likert scale was used (1 point as “strongly disagree”, 5 points as “strongly agree”), items 2, 4, 5, 8, 9, and 10 were scored in reverse. The total score of DDI is 12~60 points; higher scores indicate a greater likelihood of self-disclosure. The Chinese version of DDI has been proven to have good reliability and validity [39]. Cronbach’s α coefficient for this scale in patients with colorecatal cancer was 0.98.

#### 2.3.4. Herth Hope Index (HHI)

HHI is a 12-item scale developed by Herth to assess the level of hope [40]. Zhao et al. [41] translated the scale into Chinese and applied it among hemodialysis patients in 1999. The scale includes three dimensions: “temporality and future” (items 1, 2, 6 and 11), “positive readiness and expectancy” (items 4, 7, 10 and 12), and “interconnectedness” (items 3, 5, 8 and 9). A four-point Likert scale was used, ranging from 1 (very disagree) to 4 (very agree), items 3 and 6 were scored reversely. The total score of HHI is 12~48 points, with higher scores indicating greater hope level. The reliability and validity of the scale were good, Cronbach’s α coefficient was 0.97, and test–retest reliability was 0.91. The HHI scale was widely used in patients with chronic diseases. In the present study, the Cronbach’s α coefficient for this scale among colorectal cancer patients was 0.80.

#### 2.3.5. Perceived Social Support Scale (PSSS)

PSSS is a 12-item inventory designed by Zimet et al. [42] to measure the degree of support perceived by individuals. It comprises 12 items and 3 dimensions: family support (items 3, 4, 8 and 11), friend support (items 6, 7, 9 and 12), and others support (items 1, 2, 5 and 10). Each item is scored on a seven-point Likert scale from 1 (extremely disagree) to 7 (extremely agree), and the total score is 12~84 points, where a higher score indicates a higher level of perceived social support. Based on the original scale, Huang [43] divided the scale into two dimensions: internal family support and external family support (friend support and others support). The reliability of the scale proved to be good: Cronbach’s α coefficients of the total scale, internal family support and external family support were 0.92, 0.85, 0.91, respectively [44]. In this study, the Cronbach’s α coefficients of total scale and two subscales were greater than 0.90.

### 2.4. Statistical Analysis

SPSS (Version 26.0) and jamovi (Version 2.3) software were used for all data analyses, *p* ≤ 0.05 is considered statistically significant. The distributions of sample data were evaluated by Shapiro–Wilk tests (when *p* > 0.05, it is considered that the data are normally distributed) combined with histograms and sample sizes. Because the sample data were normally distributed or approximately normally distributed, parametric tests were applied. Sociodemographic characteristics, disease-related characteristics, levels of CRF, self-disclosure, hope, and social support were analyzed using descriptive statistics. The data were described by mean, standard deviation (SD), 95% CI, frequency, and composition ratio according to data types. Independent samples t-test and one-way analysis of variance (ANOVA) were employed to compare the differences of CRF scores in participants with different characteristics. The homogeneity of variances was verified by Levene tests. Pearson’s correlation coefficients were used to test the relationships between self-disclosure, hope, social support, and CRF. The results of correlation analyses were presented in a heatmap. Multiple linear stepwise regression analysis was conducted to determine the correlates of CRF. Statistically significant variables in univariate analyses were included in multivariable analysis. The variance inflation factor (VIF) was used to diagnose multicollinearity problems.

## 3. Results

A total of 370 colorectal cancer patients undergoing postoperative adjuvant therapy were initially enrolled, of which seven cases were excluded due to invalid data; thus, a sample of 363 patients was finally included for data analyses (effective response rate of 98.11%).

### 3.1. The Characteristics of the Sample

The 363 respondents were a mean age of 58.82 years (SD = 11.29); we grouped the age range according to the retired age. Because the current retired age in China is 60 years old, we defined 60 as a critical age level for data analysis. Most of participants were males (62.81%), married (94.49%), and unemployed (89.81%); more than half of the participants (52.89%) were under 60 years old, 52.34% had normal weight, 45.73% had elementary school and below education, and 47.11% had an average family monthly income of less than or equal to 1000 CNY. Most patients were colon cancer patients (51.24%), had a higher cancer stage (79.06%), had less than half a year since the operation (81.54%), were treated with chemotherapy only (83.20%), had no more than six chemotherapy cycles (92.56%), and had no pain (86.23%) or no sleep disorder (87.33%). More information about the sociodemographic and colorectal cancer-related characteristics of the sample are presented in Table 1.

### 3.2. Scores of CFS among Colorectal Cancer Patients

**Figure 2:**
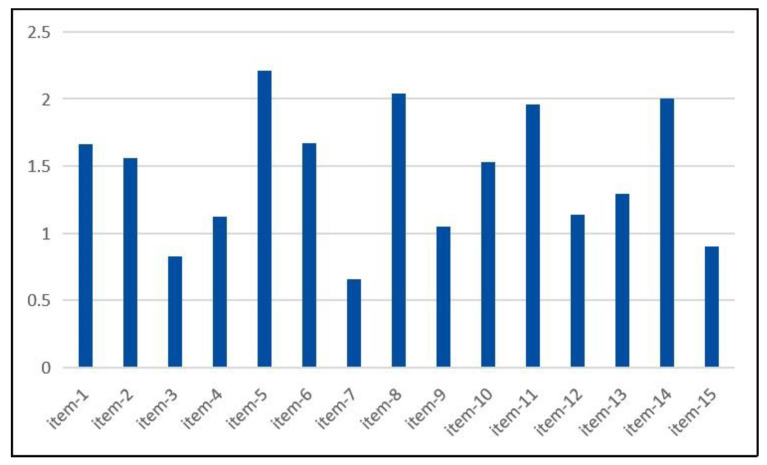
The mean score of each item of CFS. Note: CFS, cancer fatigue scale.

The incidence of fatigue among colorectal cancer patients undergoing postoperative adjuvant therapy was 100.00%. The mean score of CFS was 21.61 (SD = 6.16). The fatigue degree rating was “moderate”. Among the three dimensions of CFS, the “physical fatigue” had the highest score, and the “cognitive fatigue” yielded the lowest score; however, the “emotional fatigue” earned the highest mean score of items (e.g., “Item 5: Do you feel energetic?”, “Item 8: Do you feel interest in anything?”, “Item 14: Can you encourage yourself to do anything?”, “Item 11: Can you concentrate on certain things?”, see Figure 2). Other information about CFS scores were reported in Table 2.

### 3.3. Comparisons of CFS Score among Different Sample Groups

Univariate analyses showed that there were significant differences in CFS scores among patients with different sex, BMI, per capita monthly income, clinical stages, time since operation, treatment methods, number of chemotherapy cycles, pain, sleep disorder groups. The scores of CFS were significantly higher among patients who were females, those with a BMI of less than 18.5 kg/m^2^, those with a per capita monthly income of less than or equal to 1000 CNY; those with clinical stages III or IV, those with time since operation more than 6 months, those who underwent chemotherapy + targeted therapy/chemotherapy + immunotherapy, those with number of chemotherapy cycles more than 6 times, and those who had pain or sleep disorder. The results are shown in Table 1.

### 3.4. Correlations between CFS Score and Other Psychosocial Variables

**Figure 3:**
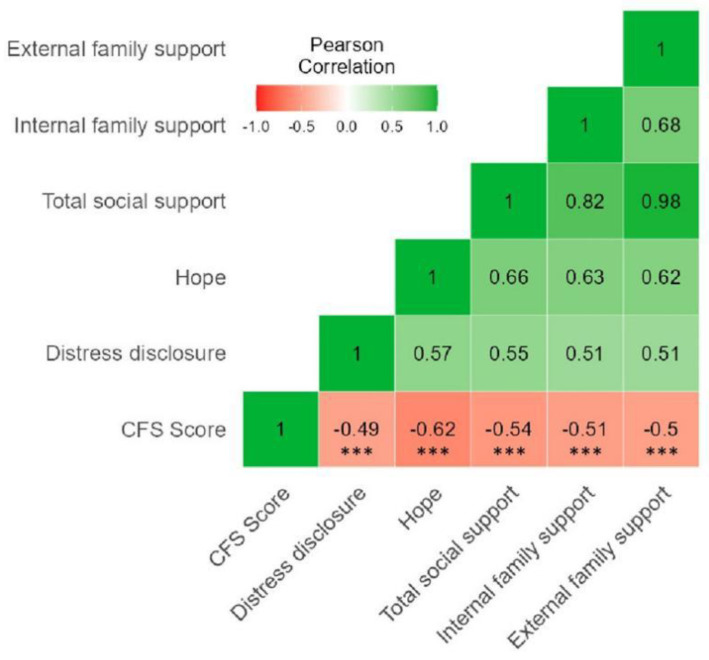
Correlations between CFS score and other psychosocial variables: a correlation heatmap. Notes: CFS, cancer fatigue scale; The number in the box **☐** represents Pearson’s correlation coefficient, positive number represents positive correlation, and negative number represents negative correlation; *** *p* < 0.001.

The scores of other psychosocial variables are presented in Table 3. Pearson’s correlation analyses showed that DDI, HHI, total social support, internal family support, external family support were significantly negatively correlated with CFS score. Among these psychosocial factors, the hope level and CFS score yielded the highest correlation (*r* = −0.62, *p* < 0.001) (see Figure 3).

### 3.5. Multiple Linear Regression Analysis

The CFS score was used as the dependent variable, the above related factors with *p* < 0.05 (sex, BMI, per capita monthly income, clinical stages, time since operation, treatment methods, number of chemotherapy cycles, pain, sleep disorder, DDI, HHI, social support) were used as independent variables. A multiple linear regression model was established, using the stepwise method to screen the independent variables (*α*_enter_ = 0.05, *α*_remove_ = 0.10).

Variance inflation factors ranged from 1.034 to 1.935 (<10), illustrating the absence of multicollinearity problems between independent variables. This model showed that HHI, sleep disorder, internal family support, DDI, pain, and time since operation were significant correlates of CRF; these variables accounted for 49.1% of the total variance in CRF. The overall model was built successfully (*F*_(6, 356)_ = 59.087, *p* < 0.001). HHI was the strongest predictor of CRF (*β* = −0.397, *p* < 0.001). The results are presented in Table 4.

### 3.6. Influencing Factors Model of CRF in Patients with Colorectal Cancer

**Figure 4:**
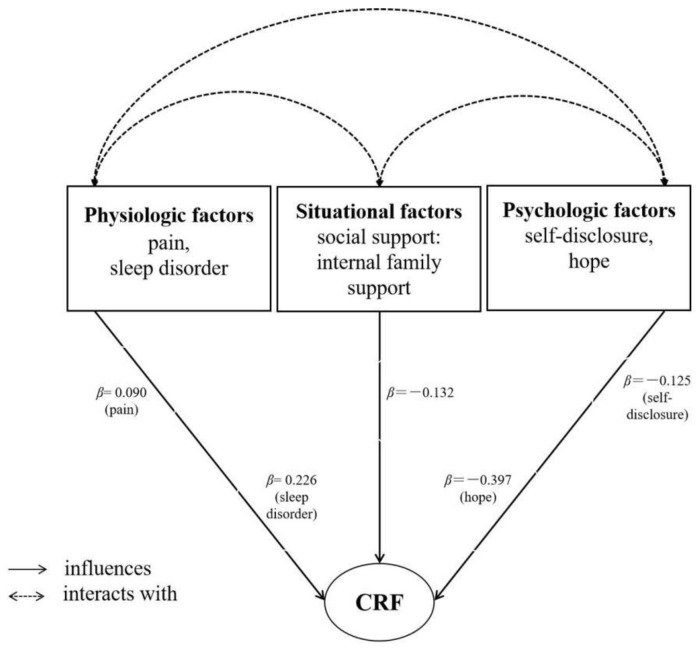
The model on modifiable influencing factors of CRF in patients with colorectal cancer. Notes: CRF, cancer-related fatigue; β, standardized coefficient.

Combined with the previous hypothesis and the findings of this study, we preliminarily constructed a model on modifiable factors of CRF among colorectal cancer patients (see Figure 4). Because the shape of this model is a bit like a “parachute”, we named it the “parachute model” of the influencing factors of CRF.

## 4. Discussion

This study was one of the beneficial attempts in evaluation of CRF among colorectal cancer patients undergoing postoperative adjuvant therapy, where we aimed to determine the physiological psychosocial correlates of CRF based on the TUS. The findings showed that the physiologic factors of pain, sleep disorder, and time since operation as well as the psychosocial factors of self-disclosure, hope level, and internal family support were significant independent predictors of CRF in this population. Furthermore, we constructed a preliminary predictive model on modifiable factors of CRF, providing a better understanding of the factors associated with CRF, and helping develop effective symptom management strategies for individuals with colorectal cancer.

The current study suggested that CRF was also a particularly frequent symptom in the majority of colorectal cancer patients during postoperative adjuvant therapy, with a relatively high prevalence. The average degree of CRF was moderate. This finding was consistent with Ouyang’s study [45]; in their report, the incidence of CRF among colorectal cancer patients was 86.6%, and the fatigue degree was from moderate to severe. As for the three dimensions of CRF, the average scores of items from high to low were emotional fatigue (2.06, 8.22/4), physical fatigue (1.26, 8.80/7), and cognitive fatigue (1.15, 4.60/4). Although the conclusion was consistent with Zou’s research [46], the average scores of items in the three dimensions were lower than their findings; in their study, the average scores of items in emotional fatigue, physical fatigue, and cognitive fatigue were 2.14 (8.55/4), 1.87 (13.07/7), 1.64 (6.57/4), respectively. This difference may be related to different types of cancer and severity of illness. The top five items (items 5, 8, 14, 11, 6) in the score ranking belonged to the dimensions of emotional fatigue and physical fatigue, suggesting that CRF had a greater impact on the emotional and physical functions of colorectal cancer patients but less on cognitive functions. The main characteristics of emotional fatigue in our sample were lack of energy, decreased interest in many things, reduced concentration when doing things, etc. The main manifestations of physical fatigue included feelings of tiredness and weakness, urgent need for rest, etc. The impacts of CRF on patients’ daily living and quality of life were substantial. Such high incidence and severity indicated the need for medical staff to strengthen the awareness and management of CRF (especially emotional fatigue and physical fatigue) in patients with colorectal cancer.

The associations between CRF and sociodemographic characteristics remain inconclusive. Take the per capita monthly income as an example; initially, we assumed that colorectal cancer patients with lower income (e.g., ≤1000 CNY) could have more fatigue. Although the result of univariate analysis showed that the CFS score was significantly higher in patients with a per capita monthly income of less than or equal to 1000 CNY than another group (*t* = 2.64, *p* = 0.009), multivariable analysis showed that the variable of per capita monthly income did not enter the regression model, suggesting that it was not a significant predictor of CRF. This conclusion is consistent with the reports of Al Maqbali [8], Ma [10], etc. but different from Shao’s report [30]. In view of the cross-sectional study design, the relationship between research factors and outcome variable is exploratory, so this causal relationship should be cautiously interpreted.

The results of regression analysis revealed that time since operation of the patients with colorectal cancer were positively correlated with CRF (*B* = 1.334, *p* = 0.028), which corresponded with prior research [11]. The CFS score of colorectal cancer patients greater than 6 months after surgery was 1.334 points higher than that of the patients within 6 months after surgery. Longer time since operation may mean longer duration of adjuvant therapy: for example, in 67 patients greater than 6 months after surgery of our study, the proportion of patients undergoing adjuvant therapy for more than 6 cycles was higher than that of patients within 6 months after surgery. Generally, patients with colorectal cancer after surgery should undergo adjuvant therapy for 6–8 cycles, approximately once a month. They completed most of their primary treatments between 6 and 12 months after surgery, during which time they had suffered a large number of adverse effects of cancer treatments. These treatments may induce or aggravate fatigue by stimulating the production of pro-inflammatory cytokines [47]. Therefore, we should pay more attention to cancer patients with longer postoperative time and more chemotherapy cycles.

Pain and sleep disorder were related to CRF based on the results of multivariable analysis (*B* = 1.614, *p* = 0.021; *B* = 4.185, *p* < 0.001; respectively), which is in line with previous study among lung cancer patients [48]; the CFS score of patients with pain or sleep disorder was 1.614 points or 4.185 points higher than that of the patients without pain or sleep disorder. Due to cancer itself and cancer treatments, patients often experience some symptom clusters; one of the most commonly identified symptom clusters is fatigue, sleep disorder, and pain [49]. These symptoms are co-occurring and interrelated: sleep disorder may affect patients’ perception and experience of CRF [50], while pain may influence CRF by limiting patients’ activity and rest as well as increasing the release of inflammatory mediators. Thus, nurses and other healthcare providers should focus on the cancer patients with pain or sleep disorder and take some pharmacological or non-pharmacological interventions to reduce these two symptoms so as to alleviate their CRF.

Self-disclosure was defined as a process of sincerely sharing personal or private thoughts and feelings with a target person [32]. To our knowledge, this study is an earlier or the first attempt to explore the relationship between self-disclosure and CRF in patients with colorectal cancer. The results of correlation analysis and regression analysis indicated that self-disclosure was a negative predictor of CRF, which was consistent with Li’s finding in ovarian cancer patients [51]. The B value for the DDI was −0.086, which meant that for every 1 point increase in self-disclosure score, the patient’s CRF score would decrease by 0.086 points. Distress disclosure includes verbal self-disclosure and written emotional disclosure. There was evidence that self-disclosure could reduce psychological burden, manage negative emotions, and ultimately promote mental health [52]. However, the condition of self-disclosure in our sample was not satisfactory, with a mean score of 37.03. Although it was slightly higher than the score of gynecological cancer patients surveyed by Song [34], it was still at a moderate level. CRF was significantly associated with the levels of distress [53]. Thus, healthcare providers should encourage patients to take appropriate ways to disclose their distress, troubles, difficulties, and obtain available emotional support, spiritual support, and material support, so as to relieve CRF.

As a concept related to positive psychology, hope was defined as “a multi-dimensional, dynamic, and empowering state of being, that is central to life, associated with external help and caring, oriented towards the future and highly personalized to each individual” [54,55]. Based on the results of univariate analysis and multivariable analysis, the hope level of patients with colorectal cancer was above the moderate level, with a mean score of 37.5; there was a significant negative correlation between the level of hope and CRF, suggesting that the higher the level of hope, the lower the score of CRF. Similarly, Shun et al. [56] also found this relationship in newly diagnosed cancer patients. For the hope index, the B value was −0.742, representing that for every 1 point increase in the hope index score, the patient’s CRF score would decrease by 0.742 points. Hope could foster psychological adjustment, positive coping skills, and self-confidence; promote positive affect; empower spiritual strength; improve anxiety, depression, exhaustion, and other psychological distressing symptoms, playing a crucial role in patients’ psychological well-being. Based on these reasons, some tailored psychological interventions, such as hope therapy, should be taken to help cancer patients improve the level of hope and reduce fatigue.

As an important external resource available to individuals, social support has a positive impact on patients’ physical function, mental health, and disease adaptability [57], and it has been proposed as an important aspect of cancer recovery, as well as in planning supportive care of cancer survivors [58]. It is a commonly used self-management method for CRF. The total score of social support in patients with colorectal cancer was 62.15, which was above the moderate level, indicating that the social support of them was good. Although the correlation analyses showed that both the internal family support and the external family support were all negatively correlated with the CRF. Interestingly, findings from the regression model demonstrated that internal family support was a stronger influencing factor of CRF (*B* = −0.380, *p* = 0.009), indicating that for every 1 point increase in internal family support, the patient’s CRF score would decrease by 0.38 points, while external family support was not statistically significant. This conclusion supported previous opinions that the most effective social support was mainly from patients’ spouses, partners, children, and other close family members (e.g., parents) [59]. During the COVID-19 pandemic, patients and their family members stayed at home most of the time and went out relatively less, which may lead to reduced external family support to some extent. This study highlights the importance of internal family support for alleviating CRF among colorectal cancer patients undergoing postoperative adjuvant therapy. However, despite the impact of COVID-19, it is necessary to further explore the effect of external family support on CRF in future studies. Social support can improve patients’ self-management ability, coping skills, and quality of life [60]. Therefore, clinicians and nurses need to further strengthen internal family support and expand external social support network of cancer patients to reduce their CRF, especially during the COVID-19 pandemic.

### 4.1. Limitations

There are some limitations that need to be noted in our study. First, this cross-sectional survey was conducted only in a single geographical area, which may weaken the representativeness of the sample to a certain extent. Second, given the potential biases in questionnaire investigation, such as recall bias, could be inevitable. Third, studies on the interactions between the influencing factors of CRF in patients with colorectal cancer are necessary to further explore the influence and mechanism of the correlates on CRF. Lastly, this kind of cross-sectional design may be insufficient to draw a definitive causal conclusion. Thus, qualitative, longitudinal research, and randomized controlled trials should also be carried out to extend and enrich our existing findings.

### 4.2. Clinical Implications

Although CRF is a common symptom affected by multifaceted factors, it has not been effectively managed in clinical settings. This study took the TUS as the theoretical guidance, and it assessed the correlates of CRF from the aspects of physiologic, psychologic, and situational factors. The results of our study provided a better understanding about the correlates of CRF among colorectal cancer patients. These findings can be used by frontline healthcare professionals for identifying the patients at high risk for CRF and developing tailored interventions to promote its management so as to improve the health prognosis of this patient population.

## 5. Conclusions

The current study explored the correlates of CRF in patients with colorectal cancer based on the TUS, and identified hope, sleep disorder, internal family support, self-disclosure, pain, and time since operation as influencing factors of CRF. Among them, hope, self-disclosure, and internal family support were the negative predictors of CRF. These findings suggest that healthcare providers could help patients alleviate CRF through psychosocial interventions such as promoting self-disclosure, enhancing social support, and improving hope level. Moreover, other interventions for controllable factors such as pain and sleep disorder are also warranted. The research on the correlates of CRF can provide an important guidance for the symptom management and treatment of colorectal cancer patients.

## Figures and Tables

**Table 1:** General characteristics of the sample, comparisons of CFS score among different sample groups (*n* = 363).

Variables	*n* (%)	CFS Score Mean (SD)	*t/F*	*p*
Sex			−3.30	0.001 **
Male	228 (62.81)	20.80 (6.05)		
Female	135 (37.19)	22.98 (6.13)		
Age (years)			0.40	0.688
<60	192 (52.89)	21.73 (6.35)		
≥60	171 (47.11)	21.47 (5.96)		
BMI (kg/m^2^)			5.22 ^#^	0.006 **
Low weight (<18.5)	28 (7.71)	25.18 (7.03)		
Normal weight (18.5 ≤ BMI < 24)	190 (52.34)	21.27 (5.91)		
Overweight (≥24)	145 (39.95)	21.37 (6.13)		
Educational level			2.62 ^#^	0.051
Elementary school and below	166 (45.73)	22.57 (6.09)		
Junior high school	113 (31.13)	20.76 (6.09)		
High school	53 (14.60)	20.55 (5.54)		
College and above	31 (8.54)	21.42 (7.24)		
Marital status			0.27	0.788
Yes (married)	343 (94.49)	21.63 (6.20)		
No (unmarried/widowed)	20 (5.51)	21.25 (5.58)		
Per capita monthly income (CNY)			2.64	0.009 **
≤1000	171 (47.11)	22.51 (6.17)		
>1000	192 (52.89)	20.81 (6.05)		
Occupational status			−1.20	0.230
Yes (employed)	37 (10.19)	20.46 (6.39)		
No (unemployed)	326 (89.81)	21.74 (6.13)		
Cancer type			0.38	0.705
Colon cancer	186 (51.24)	21.73 (6.42)		
Rectal cancer	177 (48.76)	21.49 (5.89)		
Clinical stage			−2.82	0.005 **
I–II	76 (20.94)	19.86 (5.56)		
III–IV	287 (79.06)	22.08 (6.24)		
Time since operation (months)			−2.93	0.004 **
≤6	296 (81.54)	21.17 (6.15)		
>6	67 (18.46)	23.58 (5.84)		
Treatment methods			−3.39	0.001 **
Chemotherapy	302 (83.20)	21.13 (5.93)		
Chemotherapy + targeted therapy/ Chemotherapy + immunotherapy	61 (16.80)	24.02 (6.74)		
Number of chemotherapy cycles			−2.47	0.014 *
≤6	336 (92.56)	21.39 (6.16)		
>6	27 (7.44)	24.41 (5.55)		
Pain			−4.91	<0.001 ***
Yes	50 (13.77)	25.46 (5.81)		
No	313 (86.23)	21.00 (6.00)		
Sleep disorder			−8.09	<0.001 ***
Yes	46 (12.67)	27.93 (4.86)		
No	317 (87.33)	20.69 (5.78)		

Notes: CFS, cancer fatigue scale; SD, standard deviation; BMI, body mass index; 1 CNY ≈ 0.14 USD. **^#^** *F* value; *** *p* < 0.001; ** *p* < 0.01; * *p* < 0.05.

**Table 2:** CFS scores in patients with colorectal cancer (*n* = 363).

Variables	Range	Mean (SD)	95% CI
Total CFS score	8–39	21.61 (6.16)	20.98–22.25
Physical fatigue	1–20	8.80 (3.62)	8.43–9.17
Item 1: Do you become tired easily?	0–3	1.66 (0.66)	1.59–1.72
Item 2: Do you have the urge to lie down?	0–3	1.56 (0.62)	1.50–1.63
Item 3: Do you feel exhausted?	0–4	0.83 (0.74)	0.75–0.90
Item 6: Does your body felt heavy and tired?	0–3	1.67 (0.61)	1.61–1.73
Item 9: Do you feel fed-up?	0–3	1.05 (0.74)	0.97–1.12
Item 12: Do you feel reluctant?	0–3	1.14 (0.62)	1.08–1.20
Item 15: Do you feel such fatigue that you don’t know what to do with yourself?	0–2	0.90 (0.69)	0.82–0.97
Emotional fatigue	2–13	8.22 (1.87)	8.03–8.41
Item 5: Do you feel energetic?	0–4	2.21 (0.71)	2.13–2.28
Item 8: Do you feel interest in anything?	0–4	2.04 (0.59)	1.98–2.10
Item 11: Can you concentrate on certain things?	0–4	1.96 (0.55)	1.91–2.02
Item 14: Can you encourage yourself to do anything?	0–3	2.00 (0.49)	1.95–2.05
Cognitive fatigue	0–14	4.60 (1.91)	4.40–4.79
Item 4: Do you feel you have become careless?	0–3	1.12 (0.62)	1.05–1.18
Item 7: Do you feel that you more often make errors while speaking?	0–4	0.66 (0.66)	0.59–0.73
Item 10: Do you feel you have become forgetful?	0–4	1.53 (0.63)	1.46–1.59
Item 13: Do you feel that your thinking has become slower?	0–4	1.29 (0.62)	1.23–1.36

Notes: CFS, cancer fatigue scale; SD, standard deviation; CI, confidence interval.

**Table 3:** The scores of other psychosocial variables (*n* = 363).

Variables	Range	Mean (SD)	95% CI
DDI	22–53	37.03 (8.93)	36.11–37.95
HHI	27–48	37.50 (3.30)	37.16–37.84
Temporality and future	8–16	12.46 (1.55)	12.30–12.62
Positive readiness and expectancy	9–16	12.47 (0.94)	12.38–12.57
Interconnectedness	9–16	12.57 (1.30)	12.44–12.70
PSSS	39–84	62.15 (7.45)	61.38–62.92
Internal family support	18–28	25.16 (2.14)	24.94–25.38
External family support	16–56	36.98 (5.83)	36.38–37.59

Notes: SD, standard deviation; CI, confidence interval; DDI, distress disclosure index; HHI, Herth hope index; PSSS, perceived social support scale.

**Table 4:** Multiple linear regression model on correlates of CRF (*n* = 363).

Variables	*B*	*SE*	*β*	*t*	*p*	95% CI for B	
						Lower	Upper
(Constant)	59.872	3.308	—	18.098	<0.001	53.366	66.378
HHI	−0.742	0.097	−0.397	−7.613	<0.001	−0.934	−0.551
Sleep disorder	4.185	0.729	0.226	5.743	<0.001	2.752	5.619
DDI	−0.086	0.033	−0.125	−2.635	0.009	−0.151	−0.022
Internal family support	−0.380	0.144	−0.132	−2.641	0.009	−0.663	−0.097
Pain	1.614	0.697	0.090	2.316	0.021	0.244	2.985
Time since operation (Reference group: ≤6 months)							
>6 months	1.334	0.605	0.084	2.207	0.028	0.145	2.524

Notes: Model: *F*_(6, 356)_ = 59.087, *R*^2^ = 0.499, Adjusted *R*^2^ = 0.491, *p* < 0.001. *B*, non-standardized coefficient; *SE*, standard error; *β*, standardized coefficient; CI, confidence interval; HHI, Herth hope index; DDI, distress disclosure index; CRF, cancer-related fatigue.

## Data Availability

The data that support the findings of our study are available from the corresponding author upon reasonable request. Further inquiries could be directed to the first author (S.W.).

## Supplementary Materials

The following supporting information can be downloaded at: https://www.mdpi.com/article/10.3390/curroncol29120720/s1. Table S1: STROBE Statement—Checklist of items that should be included in reports of cross-sectional studies.
